# Angiogenic factors and the risk of preeclampsia: A systematic review and meta-analysis

**DOI:** 10.18502/ijrm.v17i1.3815

**Published:** 2019-03-07

**Authors:** Yousef Veisani, Ensiyeh Jenabi, Ali Delpisheh, Salman Khazaei

**Affiliations:** ^1^Psychosocial Injuries Research Center, Ilam University of Medical Sciences, Ilam, Iran.; ^2^Pediatric Developmental Disorders Research Center, Hamadan University of Medical Sciences, Hamadan, Iran.; ^3^Department of Clinical Epidemiology, Ilam University of Medical Sciences, Ilam, Iran.; ^4^Research Center for Health Sciences, Hamadan University of Medical Sciences, Hamadan, Iran.

**Keywords:** *Angiogenic factors*, * Preeclampsia*, * sFLT-1*, * PlGF.*

## Abstract

**Background:**

The etiological nature of preeclampsia is heterogeneous. The use of biomarkers indices in early pregnancy helps to have appropriate stratification of pregnancies into high- and low risk for the purpose of choosing timely interventions.

**Objective:**

The aim of this systematic review was to determine the pathogenic role of soluble soluble fms-like tyrosine kinase-1 (sFlt-1) and placental growth factor (PlGF) in the prediction of preeclampsia in women.

**Materials and Methods:**

We performed a systematic search of the international databases including PubMed, Scopus, and Web of Science until August 2017. The quality of included studies was assessed using the Newcastle-Ottawa Scale. The primary outcome in this review was preeclampsia. The statistical heterogeneity was assessed using the X${}^{2}$ test and quantified by I${}^{2}$. Pooled effects size was obtained by random effects model. Subgroup analyses were also carried out.

**Results:**

Totally, 284 records were identified in the initial search and 15 records were finally included in the meta-analysis. The pooled odds ratios (ORs) for the association between the high level of sFlt-1 and low level of PlGF and subsequent development of preeclampsia among women were 5.20 (95% CI: 1.24–9.16) and 2.53 (95% CI: 1.33–3.75), respectively. The mean difference for sFlt-1 and PlGF in women with preeclampsia compared to controls was 1.15 (95% CI: 0.43–1.86) and –0.94 (95% CI: –1.37–0.52), respectively.

**Conclusion:**

According to the results from this meta-analysis, increased levels of sFlt-1 and reduced levels of PlGF predict the subsequent development of preeclampsia.

## 1. Introduction

Preeclampsia is a multiorgan disease process characterized by hypertension and proteinuria. The severe preeclampsia happens with epigastric pain, headache, impaired vision, impaired liver function, thrombocytopenia, kidney dysfunction, red blood cell breakdown and low blood platelet count (1, 2). Preeclampsia is associated with maternal and fetal adverse outcomes, and occurs in 2–8% of pregnancies. It is responsible for up to 15% of preterm births (3). Some risk factors may play a main role in the etiology of preeclampsia. These risk factors include diabetes mellitus, obesity, overweight, maternal age, nulliparity, high blood pressure, hypothyroidism, renal disease and family history of preeclampsia (4). The etiology for the pathogenesis of preeclampsia is yet to be fully known (5). Some studies proposed that placenta releases soluble angiogenic factors, which play a crucial role in endothelial dysfunction and development of preeclampsia-related symptoms before clinical manifestation of preeclampsia. The soluble fms-like tyrosine kinase-1 (sFlt-1) is one of these factors. It binds vascular endothelial growth factor (VEGF) A and placental growth factor (PlGF). They are the angiogenic main factors that are responsible for placental vascular development and function of mother endothelial (6). Different studies have shown that the rate of the two factors is more precise for diagnosing and prediction of preeclampsia than any of the factors alone. There is little data on sFlt1 or PlGF in pregnant women with major preeclampsia risk factors, and the majority have a small sample size. Too small a sample usually prevents the findings from being extrapolated, and significance relations may not be seen, however, the diagnose of preeclampsia based on the cut-off values of these two factors is still unknown and the results are inconsistent (6, 7).

To the best of our knowledge, no meta-analysis has been carried out regarding the effect of angiogenic factors on the risk of preeclampsia. Therefore, this meta-analysis was conducted for assessing the association between the angiogenic factors and the risk of preeclampsia.

## 2. Materials and Methods

### Data sources

**Figure 1:**
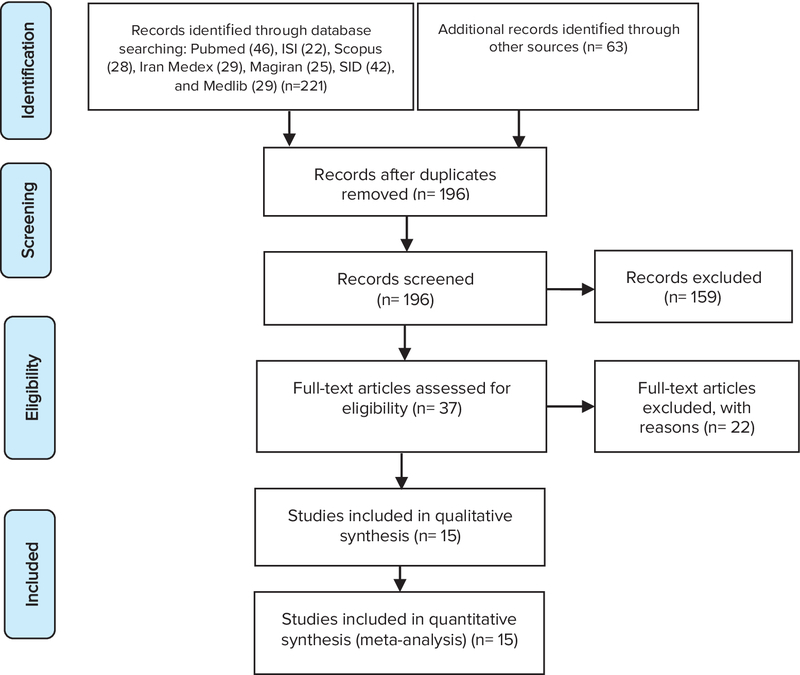
The flow diagram of selected articles in meta-analysis.

We performed a comprehensive systematic manual and electronic search to assess the pathogenic role of soluble sFlt-1 and PlGF in the prediction of preeclampsia. The PRISMA statement (8) was used to report this study (Figure 1). The relevant studies were systematically searched in international databases including Pub Med, Scopus, and Web of Science from database inception until August 2017.

### Search strategy

The search strategy was performed by the medical subject headings (MeSH) in the title, keywords, and abstracts (risks; factor; angiogenically; risk of; angiogen; pre-eclampsia; factors; angiogenicity; preeclampsia; risk; angiogenic, s-Flt-1, PLGF, biochemical blood marker; and Pregnancy) and were combined with Boolean Operators: AND and OR. After removing the duplicate articles, relevancies were checked by title and abstract review. Finally, to ensure the relevance of screened articles, their full text was reviewed. The cross-referring publication was done in order to increase the sensitivity of the initial search. There was no restriction applied on language and publication year. Among 284 selected articles in the initial search, 88 duplicate articles were removed; 159 articles rejected after reviewing abstracts, and 22 final articles remained for full-text evaluation, after these process 15 full-papers were finally selected for meta-analysis.

### Inclusion and exclusion criteria

Original researches (Case-controls and cohorts) which include these terms of examining both angiogenic factors in women with preeclampsia with two groups design, reporting of outcome information, also articles that reported the serum levels of sFlt-1 (pg per millilitre) and Free PlGF (pg per millilitre) were considered as inclusion criteria. Articles lacking reporting of the gestational age of participants and duplicate articles as well as animal studies were excluded also case reports, case series, comments, editorials, and reports were considered as exclusion criteria.

The outcome definitions were as described in the definition of PE from the International Society for the study of Hypertension in Pregnancy (9). Two investigators (S.K., E.J.) independently reviewed the full-text of articles to assess their eligibility in the meta-analysis. Any disagreement between the two was resolved by discussion within the team of authors.

### Data extraction 

After review of all searched articles (title, abstract, and full text), irrelevant studies were excluded from the study. For the studies that were eligible for meta-analysis, the following information was extracted: publication year, first author, study population, trimester of pregnancy, total sFlt-1 (pg per milliliter), Free PlGF (pg per milliliter), number of subjects in each group, standard deviation, odds ratio (OR), their associated 95% confidence intervals (CI), and study design.

### Quality assessment

Newcastle-Ottawa Scale (NOS) was used for quality assessment of the studies (10). The abstracting instrument included 8 items and 11 scores possible. Eventually, articles were classified to high quality (scoring ≥5 points) or low quality (scoring <5 points). In this meta-analysis, all the articles that obtained five or more points were included.

### Statistical analysis

The current meta-analysis was applied by two effect sizes (Mean Difference (MD) and Odds Ratio (OR)). The association of preeclampsia and angiogenic factors was reported by pooled OR. In addition, we used the MD to compare the serum level of angiogenic factors between women with preeclampsia and control group. The statistical heterogeneity among the results of included studies was investigated by X2 and quantified using I${}^{2}$. In this study, the random effects model was used when the heterogeneity was moderate to high (I${}^{2}$ > 25). Begg and Egger's tests were used to assess publication bias in included studies (11). All meta-analysis and meta-regressions were performed using Stata software version 12 (Stata Corp, College Station, TX, USA). For all effect estimates, a value of p < 0.05 was considered to be statistically significant.

## 3. Results 

Figure 1 shows the results of the literature review and selection process. A total of 284 potentially relevant articles were identified from the initial literature review (221 for major international databases and 63 for hand searching and reference lists of the identified articles). After removing the duplicates, 196 articles remained, and then the authors excluded 159 articles by screening the titles and abstracts. After full-text examination 22 articles were excluded due to improper study design or missing data. Finally, 15 studies met the eligibility criteria for meta-analysis.

Full characteristics of included studies are shown in table I. From 15 included studies, 14 studies had case-control design and one was a cohort study. All studies are published in English.

**Figure 2:**
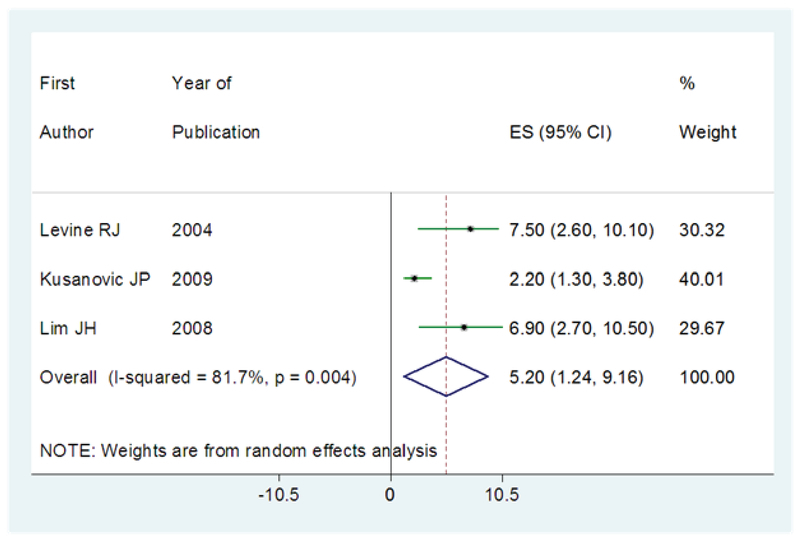
The results of meta-analysis in regard to the risk of subsequent development of preeclampsia in women with a higher value of sFlt-1 than cut-off point. The effect size is OR. Diamonds represent the overall pooled estimates of the risk of preeclampsia.

The risk of preeclampsia in women with high level of sFlt-1 was reported in three articles (12, 18, 20). The OR of the risk of subsequent development of preeclampsia in women with levels higher than the cutoff value of sFlt-1 was 5.20 (95% CI: 1.24–9.16), which means that sFlt-1 increases 5.2 times the risk of preeclampsia in cases groups. The results of I${}^{2}$ statistic showed a positive heterogeneity in articles (I${}^{2}$ = 81.7%; p ≤ 0.004) (Figure 2).

**Figure 3:**
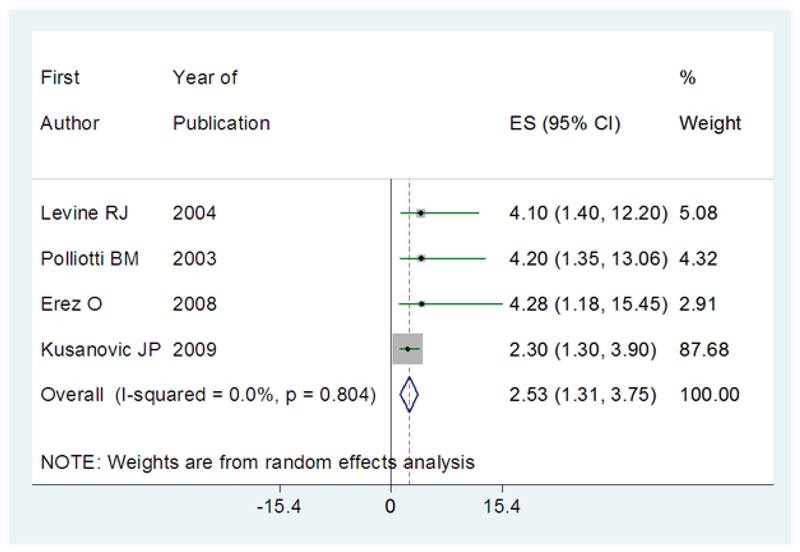
The results of meta-analysis in regard to the risk of subsequent development of preeclampsia in women with a lower value of PlGF than the cut-off point. The effect size is OR. Diamonds represent the overall pooled estimates of the risk of preeclampsia.

The risk of preeclampsia in women with low level of PlGF was reported in four articles (12, 14, 17, 18). The OR of the risk of subsequent development of preeclampsia in women with levels lower than the cut-off value of PlGF was 2.53 (95% CI: 1.33–3.75), which means that PlGF increases 2.53 times the risk of preeclampsia in cases groups. The results of I${}^{2}$ statistic did not show heterogeneity (I${}^{2}$=00.0%; p≤0.80) (Figure 3).

**Figure 4:**
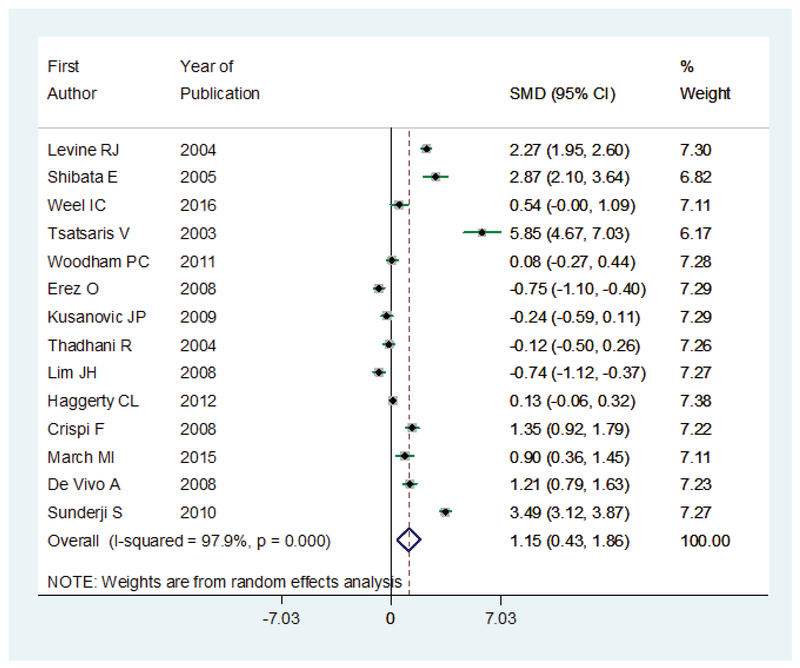
The MD for sFlt-1 in women with preeclampsia compared to controls. The effect size is MD. Diamonds represent overall pooled estimates of effects of higher sFlt-1 on preeclampsia.

Mean of sFlt-1 in 14 studies was reported. The MD was significantly increased in intervention group 1.15 (95% CI: 0.43–1.86), which concluded higher level of sFlt-1 in women with preeclampsia. The results of I${}^{2}$ statistic showed a positive heterogeneity in articles (I${}^{2}$ = 97.9%; p ≤ 0.001) (Figure 4).

**Figure 5:**
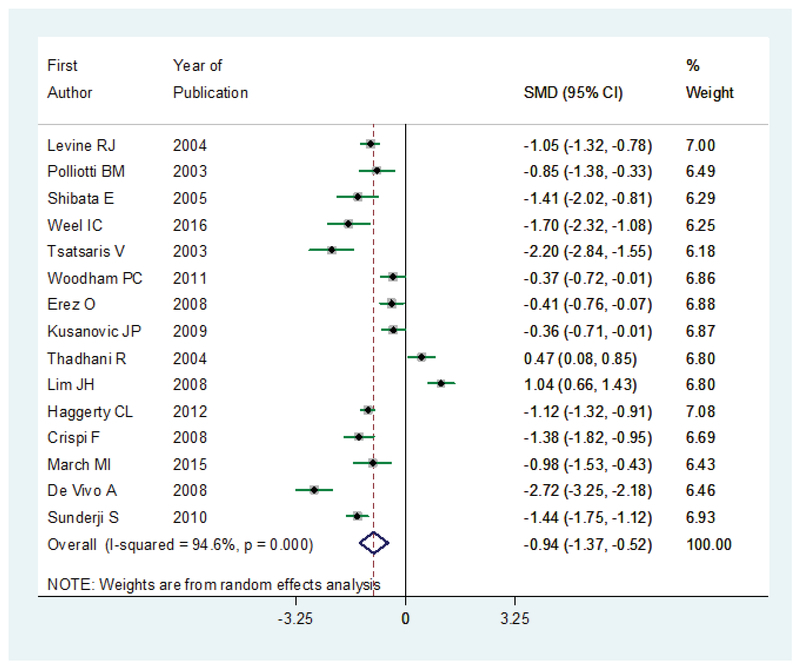
The MD for PlGF in women with preeclampsia compared to controls. The effect size is MD. Diamonds represent overall pooled estimates of effects of lower PlGF on preeclampsia.

The mean of PlGF was reported in 15 studies. The MD was significantly decreased in preeclampsia group –0.94 (95% CI:–1.37–0.52), which concluded lower level of PlGF in women with preeclampsia. The results of I${}^{2}$ statistic showed a positive heterogeneity in articles (I${}^{2}$ = 94.6%; p ≤ 0.001) (Figure 5).

In this meta-analysis, patients with preeclampsia were classified as preterm (<37 wk) or term (≥37 wk), according to the gestational age at which preeclampsia was diagnosed in enrolled studies. According to the subgroup analysis, the MD of sFlt-1 was significantly increased in intervention group 1.71 (95% CI: 0.63–2.72); therefore, patients with a higher positive change in sFlt-1 had a higher risk for the development of preterm preeclampsia. Also, patients with a low change in PlGF –1.01 (95% CI: –1.46–0.56) had a higher risk for the development of preterm preeclampsia (Table II).

**Figure 6:**
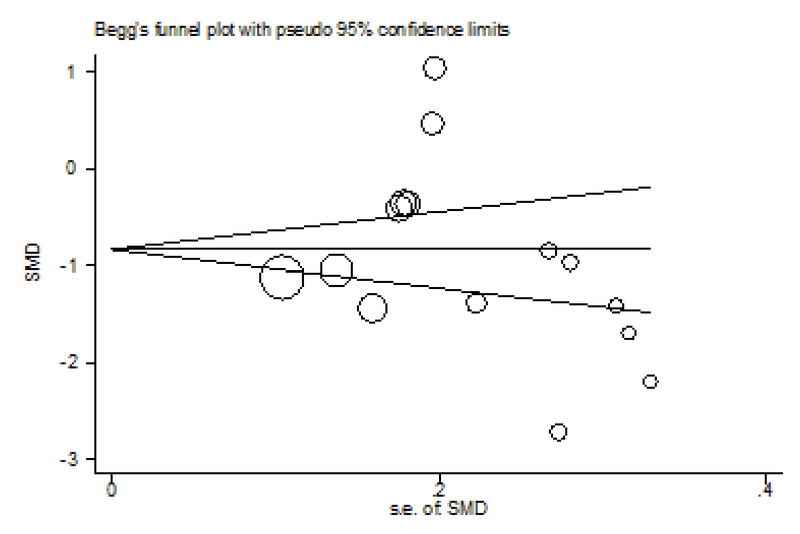
Beggs plot results for selected articles by preeclampsia between angiogenic factors and publication bias.

The possibility of publication bias was explored by Beggs test, but our results were not indicated of publication bias (Z:–0.84; p = 0.400). Therefore, we tried considering the most of published articles in this subject (Figure 6).

**Table 1:** The characteristic of included studies.


**Author (ref no)**	**Year**	**Country**	**Design**	**Women with Preeclampsia**	**Controls**	**Assessed factors**	**Gestational age**	**Quality**
Levine *et al*. (12)	2004	USA	C-C	120	120	sFlt-1, PIGF	≥ 37 Wk	High
Polliotti *et al*. (13)	2003	USA	C-C	20	60	sFlt-1, PIGF	< 34 Wk	High
Shibata *et al*. (14)	2005	USA	C-C	26	27	sFlt-1, PIGF	34–35 Wk	High
Wells *et al*. (10)	2016	Brazil	C-C	40	20	sFlt-1, PIGF	37–40 Wk	High
Tsatsaris *et al*. (15)	2003	Belgium	C-C	29	31	sFlt-1, PIGF	30–372 Wk	High
Woodham *et al*. (16)	2011	USA	C-C	41	123	sFlt-1, PIGF	≥ 37 Wk	High
Erez *et al*. (17)	2008	USA	C-C	39	201	sFlt-1, PIGF	≥ 37 Wk	High
Kusanovic *et al*. (18)	2009	USA	C	32	1560	sFlt-1, PIGF	< 37 Wk	High
Thadhani *et al*. (19)	2004	USA	C-C	40	80	sFlt-1, PIGF	1–12 Wk	High
Lim *et al*. (20)	2008	Korea	C-C	40	100	sFlt-1, PIGF	≥ 37 Wk	High
Haggerty *et al*. (21)	2012	Denmark	C-C	211	213	sFlt-1, PIGF	< 27 Wk	High
Crispi *et al*. (22)	2008	Spain	C-C	36	76	sFlt-1, PIGF	< 32 Wk	High
March *et al*. (23)	2015	Spain	C-C	24	35	sFlt-1, PIGF	> 34 Wk	High
De Vivo *et al*. (24)	2008	Italy	C-C	52	52	sFlt-1, PIGF	≥ 37 Wk	High
Sunderji *et al*. (25)	2010	USA	C-C	48	409	sFlt-1, PIGF	20–36 Wk	High
Note: C-C: case-control; C: cohort;								
sFlt-1: Soluble fms-like tyrosine kinase-1; PIGF: Placental growth factor.								

**Table 2:** Changes in the angiogenic factors (sFlt-1 and PlGF) between the preterm (< 37 weeks) or term (≥ 37 weeks) of preeclampsia.


**Angiogenic factors**		**MD (95% CI)**	**Heterogeneity**	
		**I${}^{2}$**	**p-value**
sFlt-1	< 37 Wk of gestation	1.71 (0.63, 2.72)	98.3	< 0001
	≥ 37 Wk of gestation	0.44 (–0.60, 1.48)	97.7	< 0001
PlGF	< 37 Wk of gestation	–1.01 (–1.46, –0.56)	91.6	< 0001
	≥ 37 Wk of gestation	–0.85 (–1.73,0.03)	96.8	< 0001
Note: MD: The mean difference;				
sFlt-1: Soluble fms-like tyrosine kinase 1; PlGF: Placental growth factor.				

## 4. Discussion

This meta-analysis was conducted for the assessment of pathogenic roles of sFlt-1 and PlGF to predict the risk of preeclampsia in women. According to our knowledge, this is the first meta-analysis that assessed the association between angiogenic factors and preeclampsia, by best available evidence. In this meta-analysis, for careful attention, NOS tool was used for quality assessment. The abnormalities of numerous angiogenic factors are in relation with preeclampsia, but sFlt1 and the PlGF were most studied.

According to our results, increased levels of sFlt-1 predict the risk of preeclampsia in women. Also, reduced levels of PlGF had a significant role in preeclampsia in women. Therefore, an angiogenic imbalance in pregnant woman significantly related to preeclampsia. These findings are consistent with the results of previous reports that reported increasing concentrations of sFlt-1 increased the risk of preeclampsia in women (26, 27), which occurred about five weeks before the incident of preeclampsia. The decreasing level of free PlGF and free VEGF that take place at the time of increase level of sFlt-1 to some extent may be attributable in part to the binding by sFlt-1. According to our findings, the MD of sFlt-1 was significantly increased in women with preeclampsia compared to the control groups; this rate is about 15%. Also, the MD of PlGF was significantly decreased in preeclampsia. Previous articles have reported that the decrease in the PlGF level beginning early in the second trimester is the main predictor for preeclampsia (19, 28). Haggerty *et al*. showed that low concentrations of PlGF during early gestation have a much greater risk of early-onset preeclampsia (21). Levine *et al*. showed that angiogenic imbalance is significantly to be greater in women who had early onset preeclampsia and also in pregnant women who delivered a small-for-gestational-age infant, the best explanations are defective angiogenesis in these cases (12).

According to our findings, patients with a higher positive change in sFlt-1 and also patients with a low change in PlGF had a higher risk for the development of preterm preeclampsia. On the other hand, first- to second-trimester changes in angiogenic biomarkers are predictive of preterm preeclampsia.

Currently, there is no widely accepted screening test for the prediction of preeclampsia in pregnant women. However, according to our findings, angiogenic factors can be considered as a screening tool for preeclampsia. Help in decision-making as well as the better decision in resource allocation are some other applications of our results.

There are some limitations in this meta-analysis. Firstly, due to the different inclusion and/or exclusion criteria, the heterogeneity between the studies was high; therefore, the analysis of the source of heterogeneity was limited due to different sources. Secondly, articles that were reported effects size (OR) were limited, therefore final pooled analysis was limited to four articles. Thirdly, the design of all studies was retrospective cohort and mainly case-control study, therefore results were more prone to selection bias, and information on exposure is subject to observation bias in case-control studies. Finally, data that were provided by the studies included in the meta-analysis were not sufficient to perform some subgroup analyses based on confounding variables like gestational age and other clinical subgroups.

## 5. Conclusion

In summary, our results show that the sFlt-1 concentration is five times higher in a woman with preeclampsia compared to the control groups, accompanied by a decrement in the circulating free PlGF levels. Therefore, our findings supported the results of previous reports regarding the important biologic role of angiogenic factors in preeclampsia.

##  Conflict of Interest

The authors declare that they have no conflict of interest.
